# Evaluating the relationship between RMR and Q-system for improved classification of faulted rocks and weak rocks

**DOI:** 10.1038/s41598-025-01985-1

**Published:** 2025-05-17

**Authors:** Jun-Sik Park, Young-Woo Go, Tae-Min Oh

**Affiliations:** 1 Civil & Environmental Engineering, Pusan National University, Busan, Republic of Korea; 2 Korea National Railway, Daejeon, Republic of Korea

**Keywords:** RMR, Q-system, Rock mass classification, Faulted rock, Weak rock, Tunneling, Engineering, Civil engineering

## Abstract

Rock mass classification systems play a critical role in tunnel excavation and support design by evaluating key parameters such as uniaxial compressive strength, discontinuity conditions, and groundwater conditions. To improve reliability, multiple classification systems, particularly RMR and Q-System, are often utilized together. However, existing correlation equations between RMR and Q are generally derived from datasets representing intact rock masses and may not adequately capture the geological complexities of faulted zones. This study aims to establish new site-specific correlation equations for faulted and weak rocks using geological survey data from tunnel excavation sites in the Ulsan and Gyeongju regions of South Korea, where fault zones are prevalent and classification accuracy is critical due to the proximity to nuclear infrastructure. Regression analyses yielded the equations RMR = 2.2lnQ + 22.4 for faulted rocks and RMR = 4.5lnQ + 40.9 for weak rocks, with determination coefficients (R^2^) of 0.65 and 0.48, respectively. The results confirm that existing generalized equations may fail to accurately estimate ground support requirements in faulted conditions. These findings contribute to improved classification reliability and safer tunnel support design strategies in geologically complex environments.

## Introduction

Tunneling projects inherently involve geological uncertainties due to heterogeneous and anisotropic ground conditions, which critically affect construction safety and design reliability. Accurate assessment of ground behavior is essential for mitigating geotechnical risks such as tunnel face collapse, rockfalls, and groundwater inflow. However, obtaining representative ground data during the pre-construction phase remains challenging due to site accessibility, spatial variability, and the limitations of borehole investigations.

To address these challenges, empirical classification systems—particularly the Rock Mass Rating (RMR), Q-system, and Geological Strength Index (GSI)—are widely employed to assess rock mass quality and guide excavation and support design^1,2^. These systems rely heavily on parameters such as uniaxial compressive strength (UCS), RQD, joint conditions, and groundwater. Narimani et al.^3^ emphasized that while RQD has been a core metric in rock classification for decades, its application in highly fractured or weak zones often leads to inaccurate assessments, prompting ongoing refinements using digital modeling, seismic, and machine learning techniques.

Numerous studies have attempted to correlate RMR and Q systems to improve classification efficiency and applicability. For instance, Narimani et al.^4^ proposed correlation models based on extensive datasets from igneous, sedimentary, and metamorphic rock slopes across multiple regions worldwide. Laderian and Abaspoor^5^ derived statistical relationships from more than 800 stations across 14 tunnel sites in Iran, emphasizing localized calibration. Wijaya et al.^6^ analyzed rock core data from the Meninting Dam spillway tunnel in Indonesia, developing equations tailored to poor-quality volcanic rock masses. Chaulagai and Dahal^7^ presented region-specific correlations derived from five hydropower tunnel projects spanning 14.91 km in the Nepal Himalaya. These efforts demonstrate the global interest in improving cross-system compatibility. However, previous studies consistently report that such equations are highly sensitive to regional geology and rock type. Moreover, generalized correlations often lack reliability when applied to faulted or weathered rock masses^8,9^.

Faulted zones, in particular, exhibit irregular fracturing, groundwater ingress, and mechanical degradation that severely affect tunnel stability^10,11^. In these contexts, conventional classification systems tend to underestimate support requirements, leading to overbreak, deformation, and costly redesigning. As confirmed by a previous study, the need for site-specific correlations is especially critical in structurally complex conditions.

This study addresses that gap by analyzing geotechnical data collected during excavation of tunnels in the Ulsan and Gyeongju regions of South Korea, where faulted rock masses are prevalent and construction safety holds national significance due to the proximity to nuclear infrastructure. Based on field observations, we propose refined RMR–Q correlation equations tailored to weak and faulted rock zones. The results aim to improve the accuracy of rock mass classification and contribute to more rational and adaptive support design in challenging geological environments.

## Rock mass classification

Rock mass classification systems are essential tools in geotechnical engineering, enabling engineers to assess ground conditions and select appropriate tunnel support measures. These systems categorize rock masses based on factors such as joint spacing, surface condition, groundwater conditions, and rock strength. By grouping rock masses into quality classes, engineers can predict ground behavior and optimize excavation and support strategies.

The development of rock mass classification systems began with Terzaghi^12^, who introduced a method to evaluate tunnel support requirements based on rock pressure. Lauffer^13^ later proposed the stand-up time concept, emphasizing the relationship between tunnel span and the time a tunnel remains stable without support. Reference^14^ introduced the Rock Quality Designation (RQD), which measures the percentage of core samples longer than 10 cm to quantify rock quality. RQD remains a standard index in rock engineering today. Wickham et al.^15^ introduced the Rock Structure Rating (RSR), combining structural and geotechnical factors to evaluate tunnel stability.

Two of the most widely applied systems are Rock Mass Rating (RMR) system and Q-System, both developed in the 1970s. The RMR system, developed by Bieniawski^16^ assigns numerical values based on five key parameters: uniaxial compressive strength (UCS), RQD, spacing of discontinuities, condition of discontinuities, and groundwater conditions. Discontinuity orientation is also considered as an adjustment factor. RMR values range from 0 to 100 and are divided into five classes, guiding the selection of tunnel support.

The Q-System, developed by Barton et al.^17^, evaluates six parameters: RQD, number of joint sets (*J*_*n*_), joint roughness (*J*_*r*_), degree of weathering and alteration of joints (*J*_*a*_), groundwater inflow condition (*J*_*w*_), and stress reduction factor (*SRF*). These parameters are grouped into three categories and combined to calculate the Q-value, which can range from 0.001 to 1000 (Eq. 1).

1 $$Q=\frac{RQD}{{J}_{n}}\times \frac{{J}_{r}}{{J}_{a}}\times \frac{{J}_{w}}{SRF}$$

The Q-System provides guidance on tunnel support selection based on Q-values, with lower values indicating poorer rock conditions and higher support requirements. Both RMR and Q-System have proven effective in tunnel engineering. However, each system has limitations. RMR’s reliance on simplified rating scales may overlook complex ground behaviors in weak and faulted rock masses. For example, while the RMR system assigns numerical scores to parameters such as joint spacing, condition, and groundwater inflow, these values are based on categorical thresholds that may not reflect local geological complexity. In particular, the method does not explicitly account for in-situ stress, joint orientation, joint infilling materials, or time-dependent deformation, all of which are critical in faulted or highly weathered rock masses. Additionally, the simplified scoring approach may underestimate the impact of clay seams, swelling potential, or highly variable discontinuity properties. These limitations can lead to inaccuracies in classification and inappropriate support selection if RMR is applied without supplementary methods or expert judgment. The Q-System, while comprehensive, requires subjective assessments of joint conditions and does not explicitly account for rock strength. Although Barton later introduced the Qc variant to incorporate UCS, the original Q-System remains more widely used in practice. Combining RMR and Q-System assessments is often recommended to enhance reliability, particularly in challenging ground conditions. Their complementary strengths enable engineers to cross-check evaluations and reduce uncertainty in tunnel design.

## Study area

**Fig. 1:**
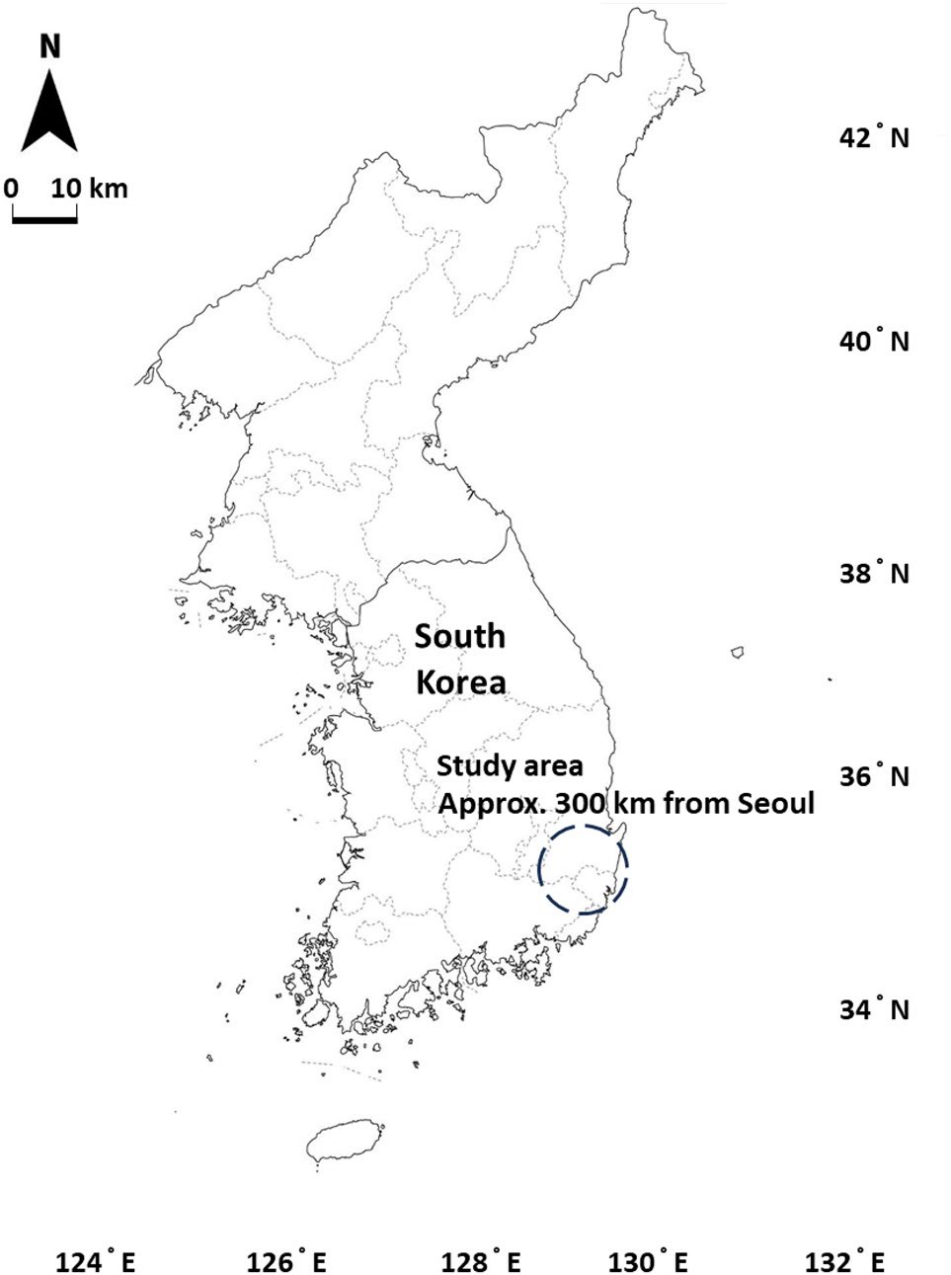
Schematic map of the study area (target site: region of Ulsan and Gyeongju).

In this study, the correlation between rock mass classification methods is analyzed in the faulted rock zones of the northern region of Ulsan and the southern region of Gyeongju (Fig. 1). This area is geographically adjacent to the seismically active Gyeongju and Pohang region in South Korea^18,19^. The proximity of this area to nuclear power plants and key national industrial complexes means that any accidents occurring during tunneling could have severe societal impacts. Given the potential risks, understanding the unique rock mass classification characteristics of this region is of paramount engineering importance for ensuring safe construction. Consequently, this study area holds significant geotechnical research value in South Korea.

**Fig. 2:**
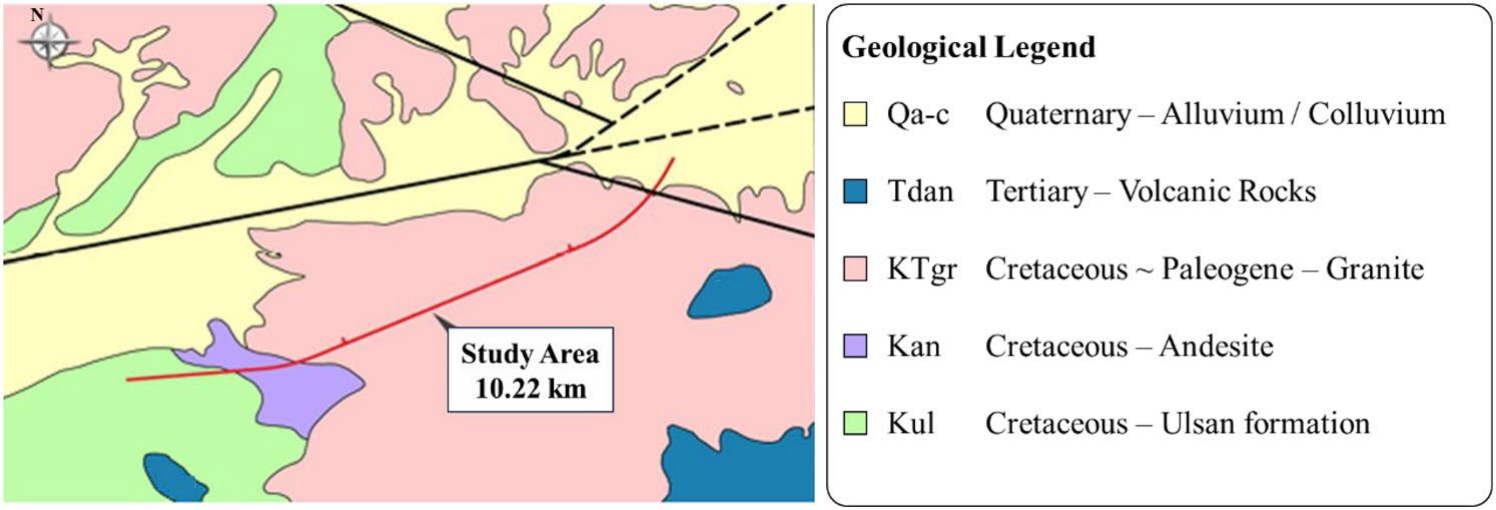
Geological map of the study area (scale 1:50,000).

The geological setting of the study area is illustrated in Fig. 2. The region primarily consists of Quaternary alluvium and colluvium, Tertiary volcanic rocks, Late Cretaceous to Paleogene granite, Cretaceous andesite, and the Ulsan Formation. These varied lithological units contribute to the complex mechanical behavior and heterogeneous ground conditions observed during tunnel excavation.

**Fig. 3:**
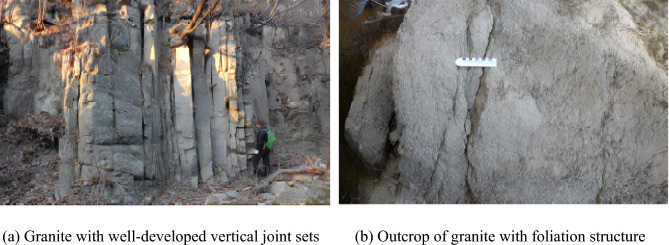
Photograph of fault zone at the target site.

**Fig. 4:**
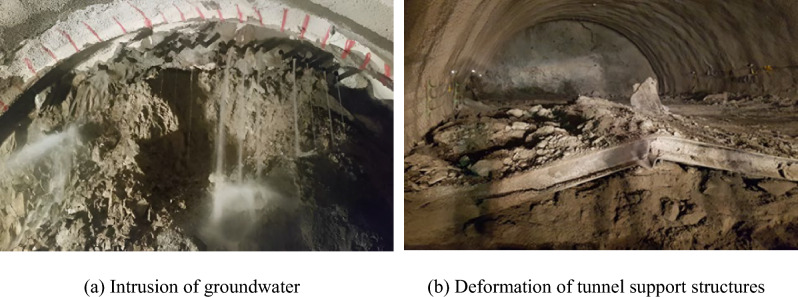
Examples of accidents during tunnel excavation.

The study area exhibits typical fault zone conditions, characterized by well-developed joints and foliation structures (Fig. 3). Faulted rocks are known for their increased permeability and mechanical instability, often leading to unexpected ground deformations, water ingress, and higher risk of collapse during tunneling. In this study, the bedrock included shale, siltstone, and granite, with a substantial weathered layer observed above the bedrock. Notably, joints were extensively developed and exhibited soil-like properties, necessitating the measurement of N values for weathered rock during geological investigations. In highly fractured zones, Rock Quality Designation (RQD) values approached 0%, reflecting extreme ground fragility. These challenging ground conditions contributed to incidents such as groundwater intrusion, roof collapse, and damage to reinforcement components during tunnel excavation (Fig. 4). In total, two major rockfalls and 68 minor collapses were reported during excavation.

Table 1 outlines six pre-designed support patterns (PD-1 to PD-6) used during tunnel construction, which were based on RMR and Q-system values. As the RMR and Q-values increased, the length of unsupported tunnel sections was extended, and the thickness of tunnel lining and shotcrete was reduced. PD-6, specifically designed for faulted rock zones, accounted for ground conditions where RMR and Q-values indicated higher instability. These support patterns were pre-designed based on past construction experience and tailored to specific terrain, geology, and tunnel cross-sections. However, as will be discussed, real-time adjustments were required during construction due to the inaccuracy of initial ground investigation results.

**Table 1:** Pre-designed tunnel support pattern of the study area.

Name	RMR	Q-system	Unsupported tunnel length (m)	Thickness of tunnel lining (m)	Thickness of shotcrete (m)
PD-1	81–100	Over 40	3.5	0.3	0.05
PD-2	61–80	10–40	2.5	0.3	0.05
PD-3	41–60	1–10	2.0	0.3	0.08
PD-4	21–40	0.1–1	1.5	0.4	0.12
PD-5	20 or below	0.1 or below	1.2	0.4	0.16–0.2
PD-6	Fault zone		1.0	0.4	0.2–0.25

**Fig. 5:**
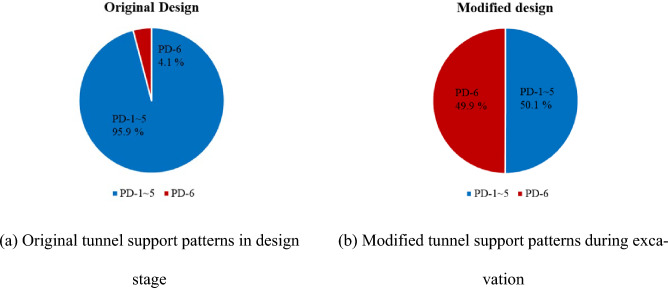
Proportion of tunnel support patterns in the study area.

Figure 5 shows proportion comparison of support patterns between original (designed before excavation) and modified (during excavation) ground support patterns. In the initial design phase for the study area, 95.9% of the total section was designed with PD-1 to PD-4 patterns, as depicted in Fig. 5a, which are typically applied to non-crushed ground. However, face mapping during the detailed design phase revealed a substantial decrease in the proportion of PD-1 to PD-5 patterns to 50.1%. Consequently, 49.9% of sections were redesigned to PD-6 (Fig. 5b).

This significant change in support design was primarily influenced by unexpected geological features encountered during excavation. While the original design relied on pre-construction borehole data that suggested relatively intact ground, detailed face mapping during excavation revealed highly fractured rock masses, fault zones, and unfavorable joint conditions that were not fully captured during the initial investigation. These findings necessitated the shift to stronger support systems such as PD-6 in nearly half of the tunnel sections.

**Fig. 6:**
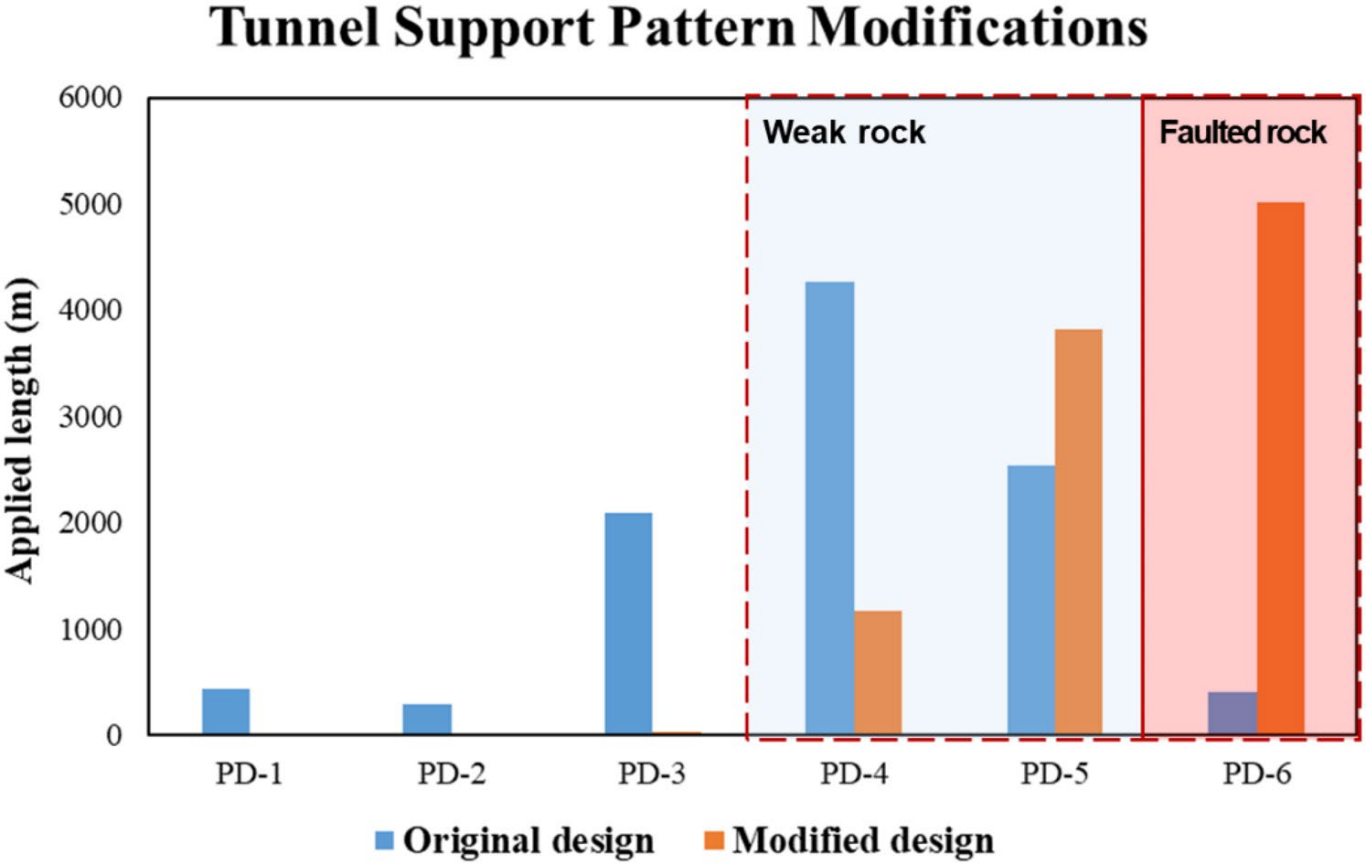
Proportion of modified tunnel support patterns in the study area.

Figure 6 presents the result of original and modified tunnel support patterns with applied length for the tunnel excavation. All tunnel construction sections were redesigned with PD-4, 5, 6. Such a substantial change in the tunnel support patterns suggests that there were significant inaccuracies in the preliminary ground investigation results. The inadequate original design of the tunnel support patterns can cause incidents such as collapses and rockfalls during the tunneling process.

Compared to previous studies that focused on generalized rock mass classification methods, this study specifically addresses the unique challenges posed by faulted rock zones. Faulted rocks are characterized by their unpredictable nature, including high levels of fracturing, water ingress, and complex stress conditions. Previous research on RMR and Q-system correlations primarily focused on more stable ground conditions, and therefore, there is a significant gap in understanding how these systems perform in challenging environments such as faulted rock zones. This study contributes new insights by showing that real-time adjustment of support patterns based on face mapping is critical in such environments, and standard preliminary ground investigations may not always be sufficient.

The findings of this study emphasize the need for flexibility in tunnel design, particularly when dealing with faulted rock zones. The real-time modification of tunnel support patterns based on face mapping allowed for successful adaptation during construction, despite the highly variable ground conditions. This approach reduced the risk of collapses and rockfalls, demonstrating that tunnel designs in complex geological settings must remain adaptable to new information gathered during excavation. This study analyzed ground investigation data collected from 106 locations throughout the tunnel excavation process. Data included compressive strength, RQD, RMR, and Q-values. These variables were selected because they represent the core parameters of the RMR and Q classification systems and are consistently quantifiable across all survey points. Although additional field observations such as weathering grade and joint infill were recorded, only the variables with standardized numeric representation were used for the correlation analysis to ensure statistical validity. The sections designed with PD-4 and PD-5 were classified as weak rock, while sections designed with PD-6 were classified as faulted rock. Correlations between these ground parameters were analyzed, providing valuable insights into the behavior of weak rock and faulted rock during tunneling. These results underline the importance of robust pre-construction investigations, supplemented by continuous real-time assessments to ensure safe and cost-effective tunneling.

## Methodology

This study adopted a multi-step approach to investigate the relationship between RMR and Q-System in faulted and weak rock masses encountered during tunnel excavation. The methodology is summarized as follows.

Initial site investigations were conducted through vertical borehole drilling along the tunnel alignment to obtain preliminary geological information. Core samples were collected and tested to estimate UCS and RQD values. In sections where poor ground conditions were expected, additional horizontal borehole drilling was carried out ahead of tunnel excavation to obtain more localized data.

During excavation, Schmidt hammer tests were performed on tunnel faces to estimate UCS. Six impact points were tested per section, and the average rebound value was used to estimate UCS. Face mapping was conducted simultaneously to evaluate discontinuities, RQD, and groundwater inflow, enabling real-time assessment of RMR and Q-values.

Data from borehole investigations and in-situ measurements were compiled and categorized into weak rock and faulted rock zones based on the applied support patterns (PD-4 and PD-5 for weak rock, PD-6 for faulted rock). Table 2 presents a summary of key geotechnical parameters categorized by support patterns. Correlation analyses were conducted to examine the relationships between UCS, RQD, RMR, and Q-values. Site-specific correlation equations for RMR and Q-values were derived for weak and faulted rock zones, considering the limitations of existing models in such challenging ground conditions. Correlation equations were derived using simple linear and logarithmic regression analyses with the coefficient of determination employed to evaluate model fit.

**Table 2:** Summary of geotechnical parameters by tunnel support pattern.

Parameters	Support pattern	Number of samples	Min–Max	Mean	Standard deviation
UCS (MPa)	PD-6	78	0–56.68	9.18	12.99
	PD-5	13	7.40–59.92	20.74	17.94
	PD-3,4	15	28.38–94.50	60.10	21.45
RQD (%)	PD-6	78	1–26	10.90	4.31
	PD-5	13	8–39	19.08	12.60
	PD-3,4	15	16–49	32.20	10.80
RMR	PD-6	78	3–19	10.47	3.40
	PD-5	13	7–30	15.45	5.82
	PD-3,4	15	23–43	30.73	5.03
Q-value	PD-6	78	0.00042–0.44	0.014	0.051
	PD-5	13	0.0088–0.80	0.10	0.22
	PD-3,4	15	0.017–0.65	0.15	0.16

## Analyses

### Characteristics of geotechnical investigation data

The uniaxial compressive strength (UCS) of the study area was obtained by two methods: the point load test and the Schmidt hammer test. During the drilling investigation at each site, point load tests were conducted on ten rock samples, and the UCS was estimated based on the average values, adjusted by a conversion factor. Subsequently, during the tunnel excavation process, Schmidt hammer tests were performed on the tunnel face of each section to estimate the UCS.

The standard conversion factor of 24 is often applied to estimate UCS from point load index (PLI) values. However, applying this uniform factor can lead to overestimation, particularly in highly fractured and weathered rock masses common in faulted zones. To address this, Kim^20^ proposed region-specific conversion factors suited to Korean rock conditions (Table 3). When the point load strength index was below 1.5, applying a factor of 24 tended to overestimate UCS significantly. In contrast, using the region-specific factors yielded UCS values that aligned more closely with Schmidt hammer estimates.

**Table 3:** Conversion factors for rock in Korea^20^.

Point load strength index (PLI, MPa)	Conversion factors	Uniaxial compressive strength ($\sigma$, MPa)
< 1.5	Do not use	< 25
1.5	15	25
1.5 < PLSI < 2.5	17 (or 6 $\times$ PLS + 5)	25 < $\sigma$ < 50
2.5	20	50
2.5 < PLSI < 6.5	22 (or 0.75 $\times$ PLS + 18.1)	50 < $\sigma$ < 150
$\ge$ 6.5	24	$\ge$ 150

**Fig. 7:**
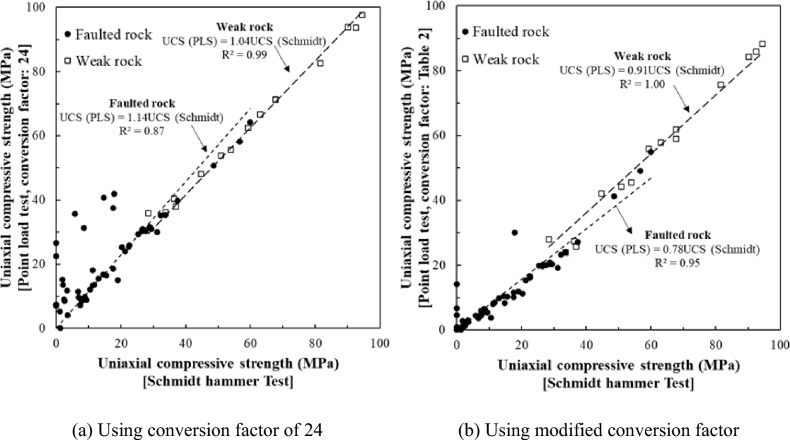
Correlation between uniaxial compressive strength estimated by Schmidt hammer test and Point load test.

Figure 7 compares UCS results from point load tests and Schmidt hammer tests. Figure 7a applies to the uniform factor of 24, while Fig. 7b uses region-specific factors. The uniform factor led to overestimation, especially in zones with UCS below 20 MPa. Region-specific factors provided more consistent estimates in highly fractured and weak rock masses, suggesting their suitability for faulted ground conditions.

**Fig. 8:**
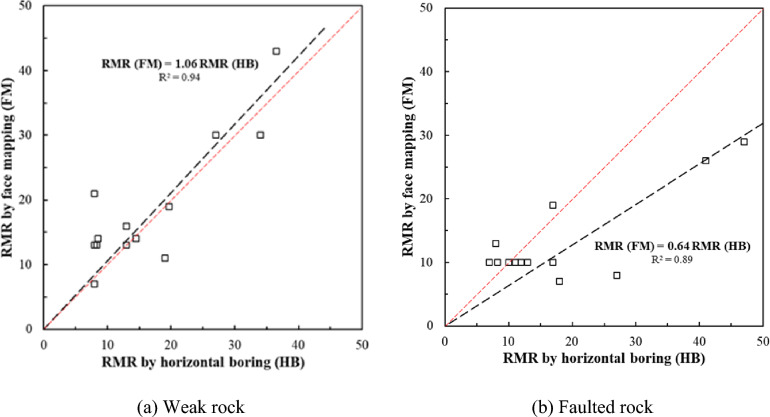
Correlation between RMR values estimated by horizontal drilling and face mapping.

Figure 8 presents the Rock Mass Rating (RMR) values obtained from both pre-horizontal drilling and face mapping. The data reveals notable differences in the RMR estimates between the two methods, especially in faulted rock zones. In weak rock conditions, the RMR values derived from horizontal drilling and face mapping were relatively consistent. However, in faulted rock zones, the RMR values obtained through horizontal drilling were found to overestimate the rock quality compared to face mapping results. This overestimation highlights a critical issue in tunnel design: relying solely on horizontal drilling data in faulted rock conditions can lead to inaccurate assessments of rock mass quality, ultimately affecting the safety and stability of the tunnel.

Face mapping provides a more reliable method for evaluating rock mass conditions in faulted rock zones, as it captures the complexity of fractured and mechanically unstable ground more accurately than horizontal drilling. This finding underscores the importance of integrating face mapping into the design process for tunnels in faulted or highly variable geological settings to ensure that support systems are appropriately designed and adjusted during excavation.

In summary, the application of uniform conversion factors in point load tests, particularly in faulted and weak rock zones, can result in overestimation of UCS, leading to potential design errors. The use of rock-specific conversion factors, such as those proposed by Kim^20^, yields more accurate results and should be considered in regions with complex geological conditions. Additionally, the discrepancy between RMR values from horizontal drilling and face mapping in faulted rock zones further emphasizes the need for accurate, real-time geological assessments to inform tunnel design and ensure construction safety.

### Correlation of rock mass classification parameters

UCS and Rock Quality Designation (RQD) are key parameters influencing rock mass classification systems like RMR and Q-System. RQD represents the percentage of intact core lengths over 10 cm and is widely used as an indicator of rock mass fragmentation. Their relationships were analyzed to assess their predictive capabilities in weak and faulted rock masses.

**Fig. 9:**
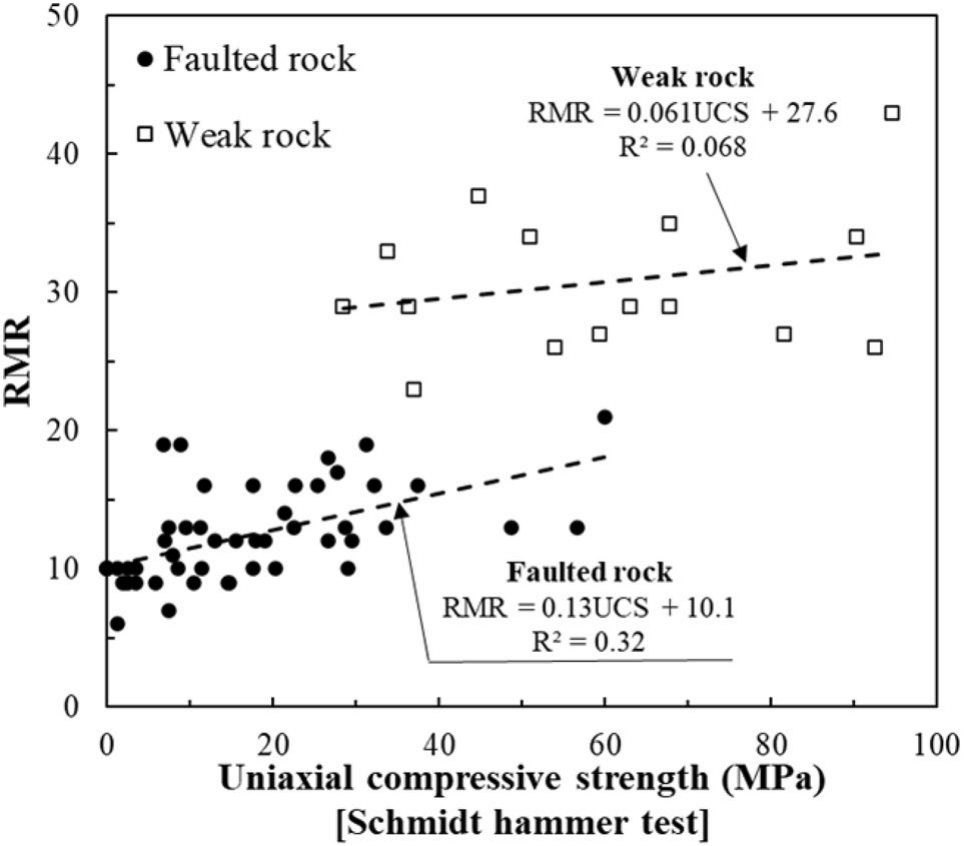
Correlation between the UCS and RMR.

Figure 9 presents the correlation between UCS and RMR. In faulted rock zones, a moderate correlation was observed, as both UCS and RMR reflect rock strength and fractured conditions. However, in weak rock zones, the correlation was weaker, suggesting that factors beyond strength, such as joint orientation and groundwater, play a greater role in determining RMR in these areas.

**Fig. 10:**
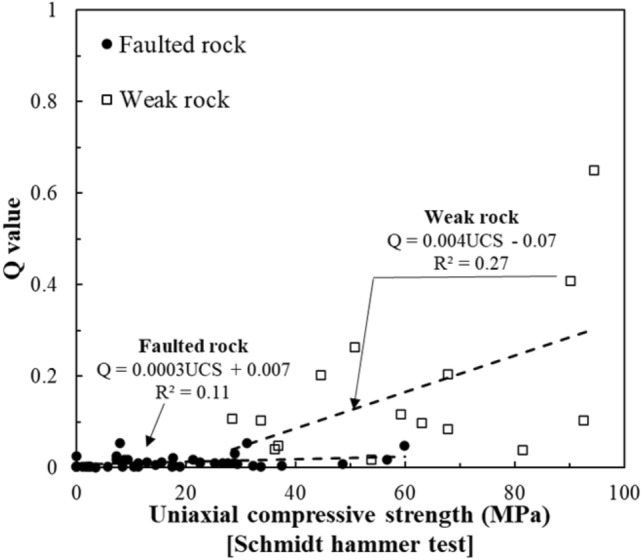
Correlation between the UCS and Q-value.

Figure 10 shows the correlation between UCS and Q-values. The relationship was weaker than UCS-RMR, particularly in faulted zones. This is because the Q-System heavily incorporates joint properties and stress conditions, which are not directly captured by UCS alone. Localized UCS measurements may be high even in heavily fractured rock masses, leading to poor predictability of Q-values.

**Fig. 11:**
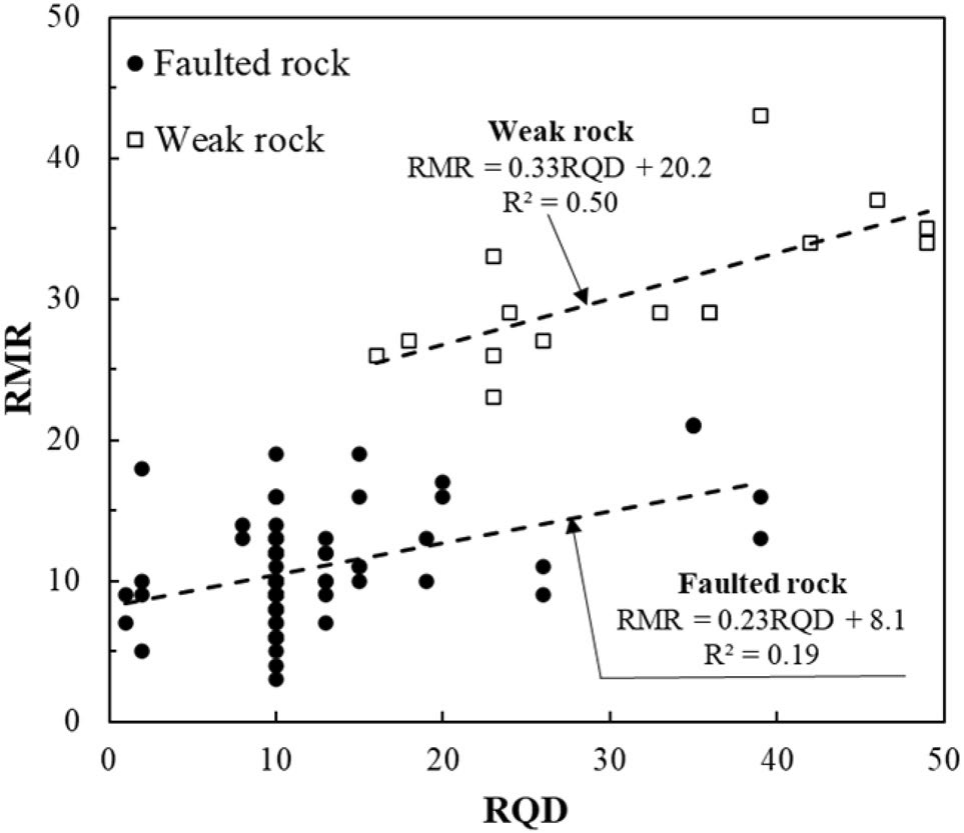
Correlation between the RQD and RMR.

Figure 11 illustrates the correlation between RQD and RMR. In weak rock zones, RQD demonstrated a relatively strong correlation with RMR, as both parameters reflect the degree of fracturing. However, in faulted rock zones, the correlation weakened. Even at low RQD values, RMR varied significantly, reflecting the influence of other factors such as joint conditions and groundwater on RMR assessment.

**Fig. 12:**
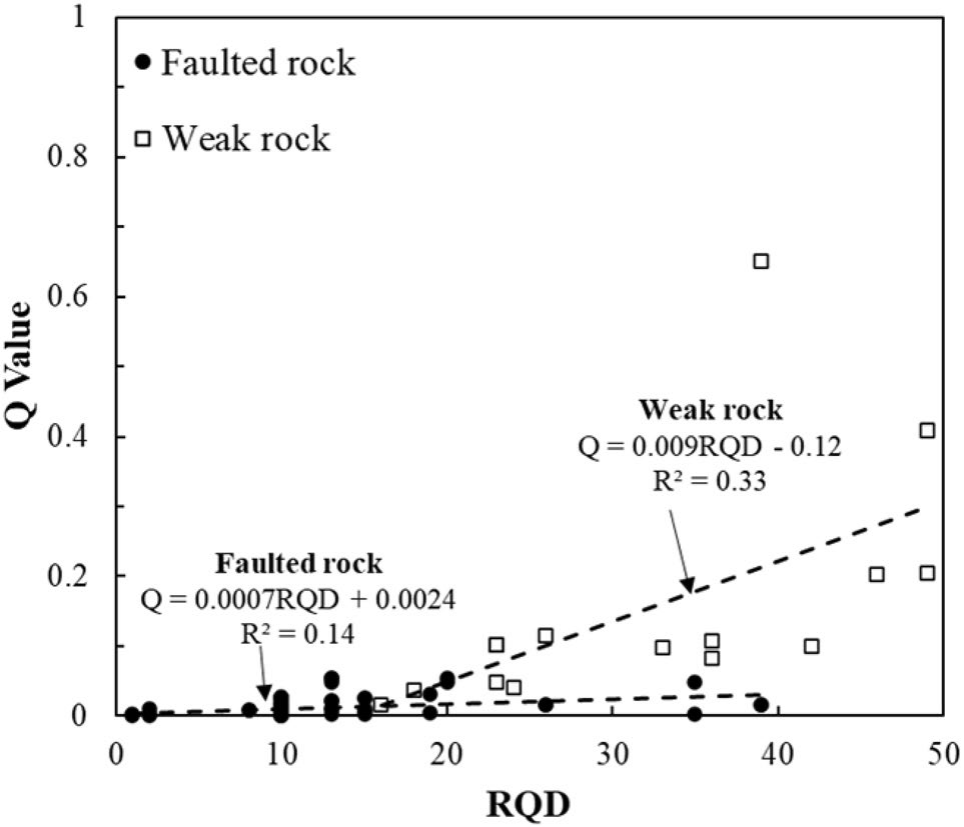
Correlation between the RQD and Q-value.

Figure 12 depicts the correlation between RQD and Q-values. In weak rock zones, a moderate positive correlation was observed. However, faulted zones displayed substantial scatter, indicating that RQD alone is insufficient to predict Q-values in fractured and water-bearing ground conditions.

The results suggest that while RQD is a valuable indicator in weak rock zones, its utility in faulted rock zones is limited. The challenging geological conditions in fault zones, such as the presence of significant discontinuities and adverse subsurface conditions (e.g., groundwater inflow), make it difficult to predict rock mass quality based solely on RQD or UCS data. Consequently, relying on geotechnical investigation data alone—such as RQD, UCS, and basic classification indices—can lead to imprecise assessments of subsurface conditions, particularly in faulted rock zones where the overall rock mass is compromised by complex geological structures.

### Correlation of RMR/Q-system

Bieniawski^21^ derived the correlation between RMR and Q-value using the Eq. (2), which is used to evaluate the reliability of RMR and Q-value obtained in tunnel construction sites. His analysis covered 111 case histories, including 68 from Scandinavia, 28 from South Africa, 12 from India, and 21 other cases from regions such as the United States, Canada, Australia, and Europe.

2 $$RMR=9\text{ln}Q+44$$

**Fig. 13:**
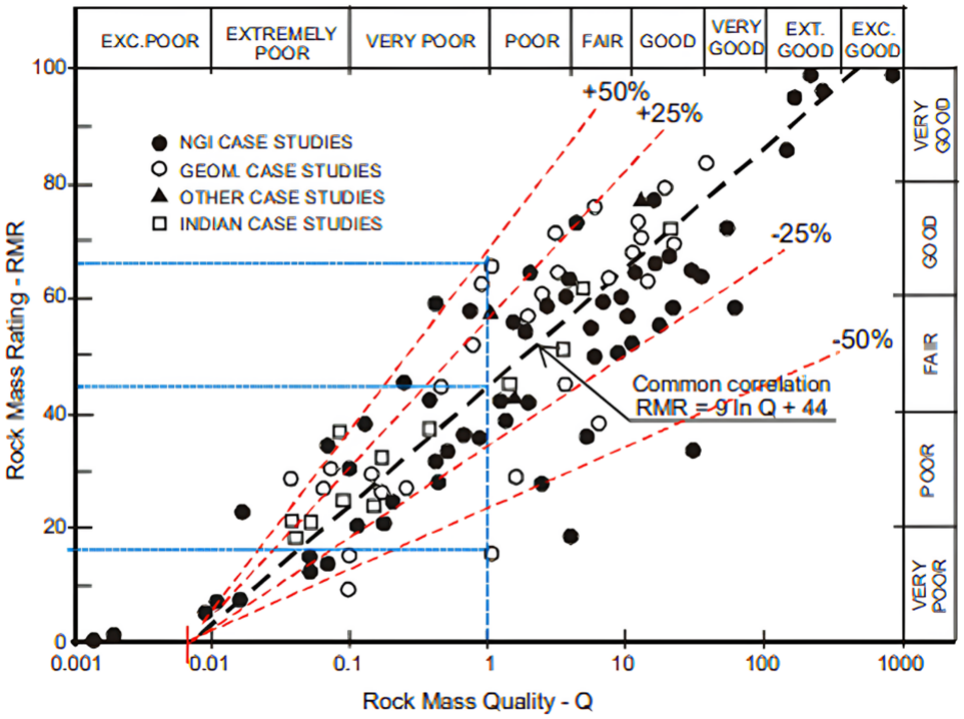
Correlation between the RMR and Q-value with deviation^8^.

However, the use of this equation results in considerable errors, with deviations of up to ± 50%. For instance, when the Q-value is 1, the corresponding RMR can range from 20 to 66 (as shown in Fig. 13). Additionally, this model becomes unreliable for Q-values less than 0.01, a range often encountered in highly fractured or faulted rock masses.

The inaccuracy of Eq. (2) stems from its inability to account for the full range of rock mass characteristics. Such errors may arise due to the limitations inherent in each classification system. For example, RMR does not fully capture ground conditions in which rock masses are severely fractured or subjected to significant in situ stress, as is common in faulted zones. Conversely, the Q-System generally provides reliable estimates for Q-values ranging from 0.1 to 40, but its accuracy diminishes outside this range^22^.

**Fig. 14:**
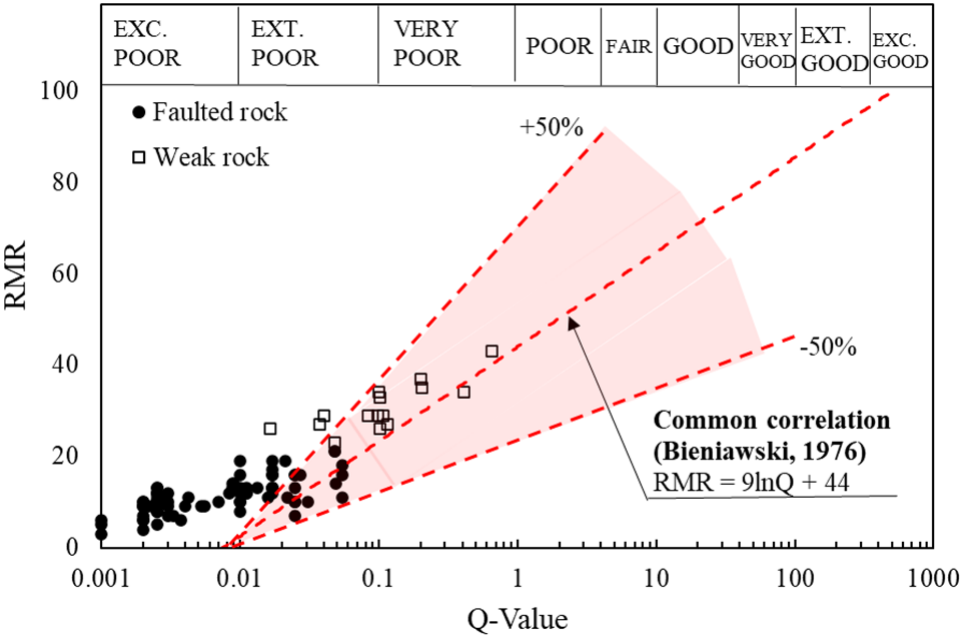
Correlation data between the RMR and Q-value with margin of error.

Figure 14 presents the distribution of RMR and Q-System data obtained from the study area, which is dominated by faulted rock zones. In these sections, most Q-values are 1 or less, with many faulted zones exhibiting Q-values below 0.1. The figure further illustrates that the data from faulted rock zones in the study area fall outside the acceptable error range for the previously established correlation equation (Eq. (2)). This discrepancy highlights the limitations of applying existing models to predict RMR in faulted rock conditions.

**Fig. 15:**
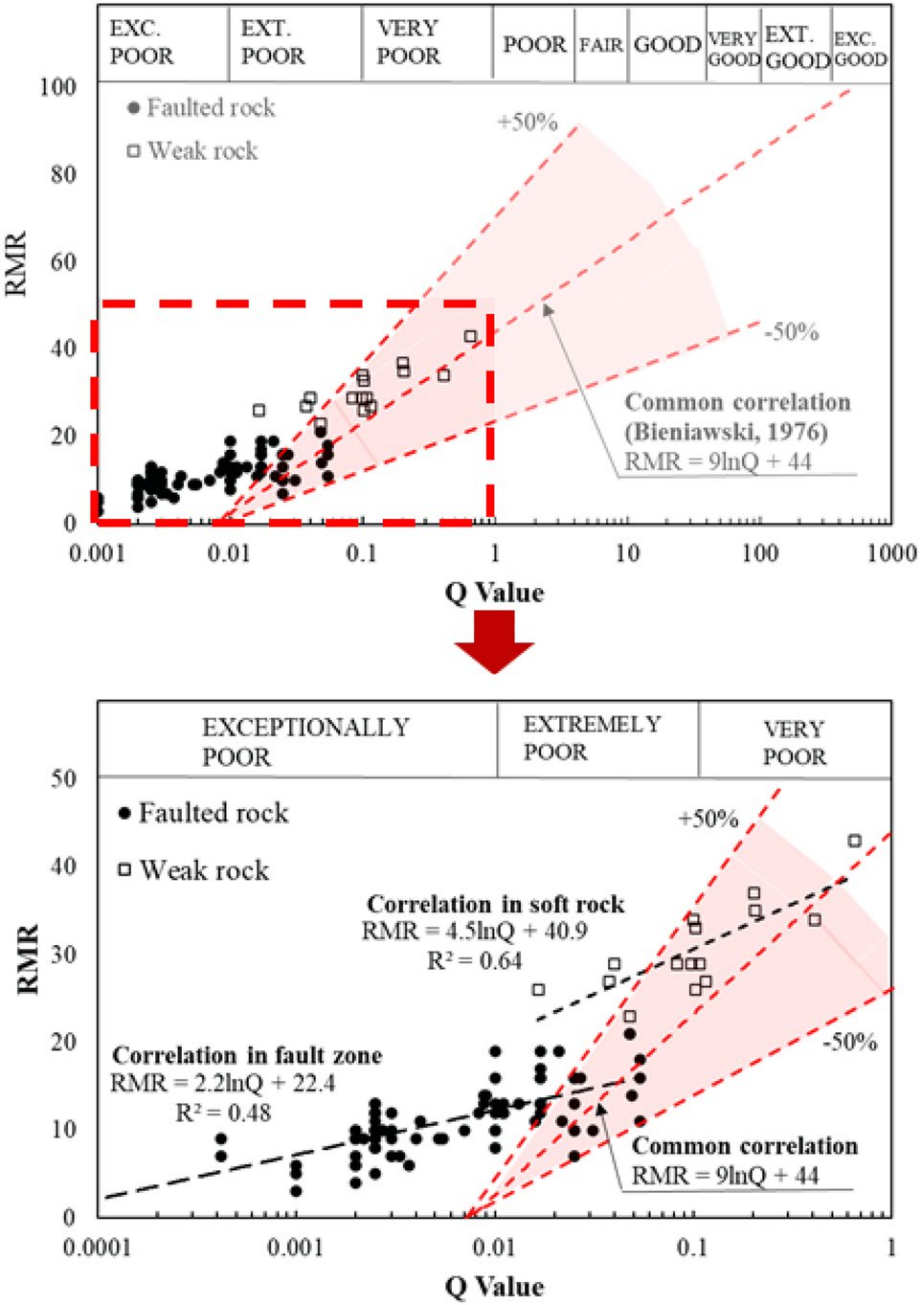
Correlation between the RMR and Q value in the study area for the faulted rock and weak rock.

To address these limitations, distinct correlation equations between RMR and Q-System are needed for the unique geological conditions of the study area. Figure 15 presents a refined correlation between RMR and Q-values, leading to the development of Eq. (3) for fault zones and Eq. (4) for weak rock zones:

3 $$RMR=2.2\text{ln}Q+22.4 (for faulted rock)$$

4 $$RMR=4.5\text{ln}Q+40.9 (for weak rock)$$

The equation for weak rock zones (Eq. (4)) closely aligns with the results from previous studies, remaining within an acceptable error range. For example, SunWoo et al.^23^ analyzed RMR–Q relationships using data collected from various tunnel construction sites across Korea, including igneous, metamorphic, and sedimentary rock formations. Their study proposed a correlation equation of RMR = 5.97 lnQ + 49.5, which shows strong similarity to the present study’s result for weak rock zones. However, the fault zone equation (Eq. (3)) differs significantly from earlier findings. This suggests that for highly fractured or faulted conditions, particularly where Q-values fall below 0.1, RMR values should be estimated more conservatively than with existing models. These findings highlight the need for more accurate correlation models, particularly in fault zones where existing equations may lead to overestimations of rock mass stability.

## Conclusions

This study aimed to refine the correlation between Rock Mass Rating (RMR) and Q-values for faulted and weak rock masses encountered during tunnel construction. By analyzing field-based geotechnical investigation data and excavation records from railway tunnels in the Ulsan and Gyeongju regions of South Korea, we developed site-specific correlation equations that more accurately represent the characteristics of structurally complex ground conditions. The key findings of this study are summarized as follows.

- The commonly used conversion factor (UCS = 24 × PLI) for estimating uniaxial compressive strength (UCS) from point load strength index was found to be unsuitable for faulted rock conditions. This uniform factor tended to overestimate UCS, especially in highly fractured zones. Applying modified, rock-specific conversion factors yielded more consistent and realistic UCS values.
- In fault zones, the correlations between UCS or RQD and rock mass classification indices (RMR and Q) were relatively weak. This reflects the complex geological behavior of such zones, where extensive fracturing, groundwater inflow, and stress-induced degradation reduce the predictive power of strength-based parameters. These findings suggest the need for cautious application of conventional classification approaches in faulted rock masses.
- A new correlation equation for faulted rock zones was derived as $RMR=2.2\text{ln}Q+22.4$ which more accurately estimates RMR in structurally degraded conditions. This equation provides a conservative and field-validated alternative to existing general models, which often overpredict RMR values and may underestimate the need for ground support.

In conclusion, this study emphasizes the importance of locally calibrated classification models in weak and faulted rock environments. The proposed correlation equation contributes to improved ground behavior prediction and safer, more adaptive tunnel support design. Future research involving additional datasets from various faulted formations will help generalize and further validate these findings.

## Data Availability

The datasets used and/or analysed during the current study are available from the corresponding author on reasonable request.
